# Prevalence and predictors of occupational burnout among first-year medical residents in Oman: the role of trait emotional intelligence

**DOI:** 10.1192/bji.2024.39

**Published:** 2025-05

**Authors:** Salim Al-Huseini, Mohammed Al Alawi, Naser Al-Balushi, Hamed Al Sinawi, Hassan Mirza, Rola Al Balushi, Manal Al Balushi, Sachin Jose, Angie Cucchi, Nasser Al-Sibani, Samir Al-Adawi, Nagina Khan

**Affiliations:** 1 Specialist Psychiatrist, Department of Psychiatry, Al Masarra Hospital, Ministry of Health, Muscat, Oman; 2 Assistant Professor and Consultant Psychiatrist, College of Medicine and Health Science, Sultan Qaboos University, Muscat, Oman; 3 Consultant Psychiatrist, Department of Behavioral Medicine, Sultan Qaboos University Hospital, Muscat, Oman; 4 Senior Consultant Psychiatrist, Department of Behavioral Medicine, Sultan Qaboos University Hospital, Muscat, Oman; 5 Clinical Psychologist, Department of Behavioral Medicine, Sultan Qaboos University Hospital, Muscat, Oman; 6 Senior Specialist Psychiatrist, Ministry of Health, Muscat, Oman; 7 Statistician, Studies and Research Section, Oman Medical Specialty Board, Muscat, Oman; 8 Senior Lecturer, London Metropolitan University, London, UK; 9 Associate Professor and Senior Consultant Psychiatrist, College of Medicine and Health Science, Sultan Qaboos University, Muscat, Oman; 10 Professor, College of Medicine and Health Science, Sultan Qaboos University, Muscat, Oman; 11 Senior Clinical Research Fellow in Primary Care, Director of Applied Health and Care Studies, Centre for Health Services Studies (CHSS), Division of Law, Society and Social Justice, School of Social Sciences, University of Kent, Canterbury, UK, email: n.khan-523@kent.ac.uk

**Keywords:** Trait emotional intelligence, abbreviated Maslach Burnout Inventory, medical residents, Oman, burnout

## Abstract

Previous research has focused on the significance of occupational burnout and the role of emotional intelligence and poor coping abilities among physicians.

Our study aimed to assess the prevalence of occupational burnout among first-year medical residents in Oman, exploring the relationship between trait emotional intelligence subscales and the three dimensions of burnout syndrome, and examining the association between sociodemographic covariates and the three dimensions of burnout syndrome.

The outcome measures included various indices of the abbreviated Maslach Burnout Inventory. The Trait Emotional Intelligence Questionnaire (TEI) and its subscales were examined.

The data showed a high burnout rate of 25.8%. Specifically, among the residents, 57.5% reported high levels of emotional exhaustion, 50.8% reported high depersonalisation and 49.2% reported a low sense of personal achievement. Age was significantly associated with depersonalisation (P < 0.003) and personal achievement (P < 0.0001). Marital status was the only variable significantly associated with emotional exhaustion (P = 0.001). Single residents had considerably lower emotional exhaustion than married residents (P = 0.001). The global mean score for the TEI was 4.77 (±0.64). A statistically significant relationship was found between personal achievement and emotional intelligence (r = 0.203, P = 0.026).

## Evidence of occupational burnout

Occupational burnout, often presented as physical, emotional and psychological stress, has been recognised to negatively affect medical professionals, both nurses and physicians.^1^ Evidence shows that burnout can compromise the required safety measures and high-quality care that healthcare professionals provide.^2^ It has also been noted in healthcare students.^3^ Strong evidence suggests that those likely to experience burnout during the early stages of their medical education are likely to experience occupational burnout throughout their medical careers.^4^ In particular, medical students are likely to experience a higher burnout rate than the general population.^5^ A recent systematic review and meta-analysis by Erschens et al^24^ highlighted that 7.0–75.2% of medical trainees self-reported having experienced burnout. Country-specific factors may explain this vast discrepancy in the prevalence of burnout, the instruments applied, and the cut-off criteria used to diagnose burnout.^6^ Various studies have been conducted with the objective of investigating the sociodemographic and risk factors associated with burnout among medical students. Understanding these factors could aid in laying the groundwork required for prevention and intervention. Rodrigues et al conducted a systematic review and meta-analysis, which included 4664 medical residents. Their results suggest that there is a subspecialty-specific tendency to report burnout.^6^

Conversely, some studies have found that physicians are not equally susceptible to burnout. Instead, sociodemographic factors, such as age, experience, specialisation, gender and marital status, have been noted to influence the risk of burnout.^7^ Despite the evidence reporting that sociodemographic factors play a role in burnout, some studies have stated the opposite. A systematic review focusing on burnout risk factors among European healthcare professionals^8^ concluded that sociodemographic factors have little effect on burnout. Therefore, more studies are required to identify who is more susceptible to burnout syndrome among medical professionals. Some initiatives have begun to mitigate occupational burnout among medical professionals, often tailored to specific individuals and organisations.^2,9^ Numerous interventions have been used to relieve burnout, including techniques that originate from Eastern philosophy, techniques that echo those implemented in the 1960s, alternative and complementary medicines, and lifestyle changes.^10,11^ According to the current literature, there is no substantial evidence to conclude that these attempted interventions are effective.^2,12^ Moreover, there are debates about whether there will be a remedy for burnout among medical professionals.^13^ Understanding variables that moderate and mediate burnout is vital to understanding this condition's trajectory.

Several studies have highlighted that certain temperaments strongly moderate and mediate the development and outcomes.^14,15^ One example is emotional intelligence. There is controversy about whether emotional intelligence is an ability or a dispositional tendency.^16^ Emotional intelligence as an ability has been the focus of research into how it correlates with well-being.^17^ As a dispositional tendency, emotional intelligence has not been the subject of many studies, with a few exceptions.^18–20^

More studies are required to explore whether emotional intelligence as a dispositional tendency strongly moderates and mediates burnout trajectories.^14,15^ Taking into account the existing research, it is hypothesised that there is a correlation between occupational burnout and emotional intelligence. More specifically, a higher emotional intelligence score correlates with a lower occupational burnout score. This study aims to assess the prevalence of occupational burnout among first-year medical residents in Oman, explore the relationship between trait emotional intelligence subscales and the three dimensions of burnout syndrome, and examine the association between sociodemographic covariates and burnout syndrome across the three dimensions.

## Method

### Study design, site and duration

This cross-sectional study was conducted between June and August 2018, among first-year residents enrolled in the Oman Medical Specialty Board (OMSB) training programmes. In Oman, physicians can enrol in a 5-year residency programme after completing their internship.^18^

### Setting

The participants were medical residents of the OMSB. The OMSB is an independent ACGME-accredited educational body in Muscat, Oman. Further information on the OMSB residency programme has been detailed elsewhere.^18^ The residency programmes include medical subspecialties (e.g. anaesthesia, dermatology, emergency medicine, internal medicine, radiology, psychiatry and paediatrics), surgical subspecialties (e.g. ear, nose and throat, general surgery, ophthalmology, oral and maxillofacial surgery, obstetrics and gynaecology, and orthopaedics) and laboratory subspecialties (e.g. biochemistry, haematology, histopathology and microbiology).

### Data collection and sampling methods

Participants were approached and recruited during an OMSB workshop and a teaching programme at the start of the first year of residency, before any significant clinical exposure. A stratified random sampling procedure was adopted to ensure the research sample was representative. Thus, participants were stratified according to intakes for each specialty (around 65% medical, 20% surgical and 15% laboratory). Randomisation software was used to fulfil the objective of the study. Participants were contacted to explain the objective of the present study and obtain electronic consent for participation. Participants recruited were informed that their participation was completely anonymous and voluntary. They were also informed of their right to withdraw from the study at any time. In the event of refusal to participate, replacements were found using another round of randomisation.

### Population and sample size

The study targeted all eligible first-year residents enrolled in OMSB training programmes, with the aim of addressing the expected prevalence of burnout among resident physicians in Oman (16 600 per 100 000, according to a previous study).^21^ To detect the expected difference in burnout prevalence with an alpha error of 0.1%, a power of 90% and accounting for a drop-out rate of 10%, a minimum sample size of 115 participants was required. A significance level of *P* < 0.05 was used for all statistical analyses. A total of 130 residents were approached, and 122 responded, resulting in a response rate of 93.8%. Stratified random sampling ensured proportional representation across the medical subspecialties (65%), surgical (20%) and laboratory (15%).

### Study variables

The variables collected comprised sociodemographic information, indices for soliciting emotional intelligence traits and occupational burnout. Sociodemographic information included variables such as age, gender and marital status. Socioeconomic status was obtained by questioning the type of housing (rented versus owned) and perceived monthly income (average income, high income, etc.). Participants were asked whether they attended government or private schools. Finally, they were asked to state their residency programme (medical, surgical or diagnostic). Study variables also included self-reported burnout and emotional intelligence checklists, as described below.

### Occupational burnout

Occupational burnout was measured using the abbreviated Maslach Burnout Inventory (aMBI), which consists of nine items. The aMBI is publicly available (https://www.mdpi.com/2077-0383/9/1/61/). The aMBI seven-point Likert scale (‘everyday’ to ‘never’) is divided into three subscales: emotional exhaustion, depersonalisation and personal accomplishment. The aMBI is based on three factors previously established to cause burnout among physicians.^22^ Although higher emotional exhaustion and depersonalisation scores indicate higher levels of burnout, a higher personal achievement score denotes lower levels. The aMBI has been used among different populations and its psychometric properties are adequate.^5^ Internal reliability scores of each subscale revealed the following Cronbach alpha coefficients: emotional exhaustion, α = 0.74; depersonalisation, α = 0.71 and personal achievement, α = 0.72. The composite total score of aMBI also achieved adequate internal reliability (α = 0.81). Burnout was taken as the sum of scores for each subscale. A score of 0–9 in the emotional exhaustion and depersonalisation subscales was categorised as no to low levels of burnout, whereas a score of 10–18 was regarded as moderate to severe burnout. A higher personal achievement score (10–18) indicated lower levels of burnout.

### Trait emotional intelligence

The dispositional tendency for emotional intelligence was measured using the Short Form Trait Emotional Intelligence Questionnaire (TEIQue-SF),^23^ which has a seven-point Likert scale (‘strongly disagree’ to ‘strongly agree’). The TEIQue-SF is a self-administered checklist with four subscales: sociability, emotionality, self-control and well-being. The TEIQue-SF consists of 30 items, and scores range from 30 to 210. A higher score implies a higher emotional intelligence trait.^18^ The reliability and validity of TEIQue-SF have been established among Omani residents in a previous study by Al Huseini et al.^18^ The reliability of the internal consistency of the four factors was as follows: α = 0.70 for emotionality, α = 0.80 for self-control, α = 0.78 for well-being and α = 0.75 for sociability.

### Ethical approval

Ethical approval was obtained from the OMSB Research and Ethics Committee of the College of Medicine and Health Sciences at Sultan Qaboos University, Muscat, Oman (approval number MREC#1058). Participants received information about the study and required written informed consent before proceeding. The study procedures for human experiments were carried out in accordance with the Code of Ethics of the World Medical Association (Declaration of Helsinki).

### Data analysis

Statistical analysis was performed with SPSS version 24.0 for Windows (IBM Corp., Armonk, New York, USA). Categorical data such as age and gender were summarised with counts and percentages. Mean and s.d. were calculated for continuous variables. The continuous variables were tested for normality by using the Kolmogorov–Smirnov test. The correlation of the total emotional intelligence score with the various burnout components (emotional exhaustion, depersonalisation and personal achievement) was assessed with the Pearson's correlation coefficient. The association of sociodemographic characteristics with the various components of burnout and overall burnout was tested with the Fisher's exact test and the likelihood ratio test. A multivariate binary logistic regression analysis was performed to determine the independent predictors of overall burnout. A *P*-value of <0.05 was considered statistically significant.

## Results

Table 1 summarises the demographic variables. The forms were handed out to 130 residents, with 122 returning the forms (response rate of 93.8%). Some forms still needed to be completed, so the final count was 120. Of these, 92 were women (76.7%), and the majority of the participants were married (*n* = 69; 57.5%). The mean age of the cohort was 27.03 ± 1.64 years. Most participants (84.2%) lived in their own homes, whereas 15.8% were in rented accommodation. The vast majority (85.8%) of the participants reported average incomes.

**Table 1:** Sociodemographic characteristics of the residents included in this study from 17 different subspecialities in Oman (*N* = 120)

Variable		*n* (%)
Age, years	Mean ± s.d.	27.03 ± 1.639
Gender	Male	28 (23.3)
	Female	92 (76.7)
Marital status	Married	69 (57.5)
	Single	51 (42.5)
Family house	Rented	19 (15.8)
	Owned	101 (84.2)
Perceived income	Average income	104 (86.7)
	High income	16 (13.3)
Urban–rural dichotomy	Urban	37 (30.8)
	Rural	83 (69.2)
Type of school attended	Private	6 (5.0)
	Government	114 (95.0)
Residency programme	Medical subspecialties	84 (70.0)
	Surgical subspecialties	26 (21.7)
	Diagnostic subspecialties	10 (8.3)

### Trait emotional intelligence and occupational burnout

Table 2 shows the mean (±s.d.) scores of the trait emotional intelligence score by its factors, for each subscale: well-being, 5.15 (±0.93); self-control, 4.47 (±0.80); emotionality, 4.70 (±0.79) and sociability, 4.71 (±0.79). The global trait emotional intelligence of all residents was 4.77 (±0.64). The prevalence of depersonalisation among first-year residents in this study was 50.8% (*n* = 61), and 55% (*n* = 66) had high emotional exhaustion, whereas 46.7% (*n* = 56) had a low sense of personal achievement. The overall prevalence of burnout in this study was 25.8% (*n* = 31).

**Table 2:** Mean descriptive analysis of the trait emotional intelligence and the abbreviated Maslach Burnout Inventory

		Mean (±s.d.)	Minimum	Maximum
Subscale of TEI	Well-being	5.15 (0.93)	2.50	7.00
	Self-control	4.47 (0.80)	2.50	6.33
	Emotionality	4.70 (0.79)	2.25	6.63
	Sociability	4.71 (0.79)	3.00	6.83
	Overall	4.77 (0.64)	2.77	6.37
		Mean (±s.d.)	*n* (%)	
Subscale of aMBI	Emotional exhaustion	3.53 (1.32)	66 (55)	
	Depersonalisation	3.34 (1.57)	61 (50.8)	
	Personal achievement	3.33 (1.31)	56 (46.6)	
	Overall	3.40 (0.84)	31 (25.8)	

TEI, trait emotional intelligence; aMBI, abbreviated Maslach Burnout Inventory.

### Demographic factors associated with occupational burnout

Table 3 shows the univariate analysis for subscale burnout associated with the sociodemographic factors. Regarding demographic variables, significant associations were found between age and the depersonalisation and personal achievement subscales (*P* = 0.003 and *P* = 0.0001, respectively). Marital status was the only variable associated with emotional exhaustion (*P* = 0.001). Emotional exhaustion was significantly lower among single residents (37.3%) than among married residents (68.1%). None of the other categorical variables were significantly associated with emotional exhaustion, depersonalisation or personal achievement.

**Table 3:** Association between sociodemographic characteristics and burnout domains derived from abbreviated Maslach Burnout Inventory

Variable		Emotional exhaustion			Depersonalisation			Personal achievement		
		No to low burnout	Moderate to severe burnout		No to low burnout	Moderate to severe burnout		No to low burnout	Moderate to severe burnout	
		*n* (%)	*n* (%)	*P-*value	*n* (%)	*n* (%)	*P-*value	*n* (%)	*n* (%)	*P-*value
Gender	Male	13 (46.4)	15 (53.6)	1.000	16 (57.1)	12 (42.9)	0.391	13 (46.4)	15 (53.6)	0.517
	Female	41 (44.6)	51 (55.4)		43 (46.7)	49 (53.3)		51 (55.4)	41 (44.6)	
Age, years mean ± s.d.		26.74 ± 1.57	27.27 ± 1.67	0.077	26.59 ± 1.46	27.46 ± 1.70	0.003*	26.55 ± 1.43	27.59 ± 1.70	0.0001*
Marital status	Married	22 (31.9)	47 (68.1)	0.001*	32 (46.4)	37 (53.6)	0.580	35 (50.7)	34 (49.3)	0.580
	Single	32 (62.7)	19 (37.3)		27 (52.9)	24 (47.1)		29 (56.9)	22 (43.1)	
Housing	Rented	7 (36.8)	12 (63.2)	0.464	7 (36.8)	12 (63.2)	0.319	11 (57.9)	8 (42.1)	0.803
	Owned	47 (46.5)	54 (53.5)		52 (51.5)	49 (48.5)		53 (52.5)	48 (47.5)	
Perceived income	Average income	50 (48.1)	54 (51.9)	0.108	53 (51.0)	51 (49.0)	0.422	57 (54.8)	47 (45.2)	0.433
	High income	4 (25.0)	12 (75.0)		6 (37.5)	10 (62.5)		7 (43.8)	9 (56.3)	
Urban–rural dichotomy	Urban	20 (54.1)	17 (45.9)	0.234	18 (48.6)	19 (51.4)	1.000	18 (48.6)	19 (51.4)	0.555
	Rural	34 (41.0)	49 (59.0)		41 (49.4)	42 (50.6)		46 (55.4)	37 (44.6)	
Type of school attended	Private	2 (33.3)	4 (66.7)	0.689	4 (66.7)	2 (33.3)	0.435	2 (33.3)	4 (66.7)	0.416
	Government	52 (45.6)	62 (54.4)		55 (48.2)	59 (51.8)		62 (54.4)	52 (45.6)	
Residency programme	Medical	37 (44.0)	47 (56.0)	0.603	42 (50.0)	42 (50.0)	0.222	47 (56.0)	37 (44.0)	0.666
	Surgical	11 (42.3)	15 (57.7)		10 (38.5)	16 (61.5)		12 (46.2)	14 (53.8)	
	Diagnostic	6 (60.0)	4 (40.0)		7 (70.0)	3 (30.0)		5 (50.0)	5 (50.0)	

* Statistically significant test: Fisher's exact test, likelihood ratio. *P*-value <0.05 is significant.

Table 4 shows the multivariate binary logistic regression analysis to identify the independent predictors of overall resident burnout. We found that four variables were statistically significant predictors of overall burnout. They were age, marital status, family status and type of residency programme. There was no association between global emotional intelligence and overall burnout. Overall burnout increased with increasing age (odds ratio 1.670, 95% CI 1.192–2.340, *P* = 0.003). Married residents had four times increased risk of burnout compared with single residents (odds ratio 4.660, 95% CI 1.553–13.985, *P* = 0.006). Surprisingly, the high-income group showed five times increased risk of burnout when compared with the average income group (odds ratio 5.865, 95% CI 1.357–25.348, *P* = 0.018). This might be because the number of residents in the high-income group was only 16 out of the total 120 residents. The analysis also showed that burnout was three times more common in surgical group residents than medical group residents (odds ratio 3.653, 95% CI 1.067–12.513, *P* = 0.039).

**Table 4:** Multivariate binary logistic regression analysis to identify the independent predictors of overall burnout among first-year residents

Variable	B	Significance	Odds ratio	95% CI for odds ratio	
				Lower	Upper
Global emotional intelligence	−0.027	0.254	0.973	0.929	1.020
Age	0.513	0.003*	1.670	1.192	2.340
Marital status					
Single, reference					
Married	1.539	0.006*	4.660	1.553	13.985
Gender					
Female, reference					
Male	−1.088	0.128	0.337	0.083	1.369
Family house					
Owned, reference					
Rented	−1.117	0.159	0.327	0.069	1.547
Family status					
Average income, reference					
High income	1.769	0.018*	5.865	1.357	25.348
Place of secondary school					
Outside Muscat, reference					
Muscat	−0.504	0.419	0.604	0.178	2.052
Type of school					
Government, reference					
Private	−0.656	0.629	0.519	0.036	7.411
Residency programme					
Medical, reference					
Surgical	1.296	0.039*	3.653	1.067	12.513
Diagnostic	0.219	0.815	1.245	0.199	7.797

Overall burnout defined as high emotional exhaustion plus high depersonalisation plus low personal achievement.

* Statistically significant. *P*-value <0.05 is significant.

### Relationship between trait emotional intelligence and occupational burnout

The correlation between trait emotional intelligence and various burnout components is shown in Table 5. Significant positive correlations were found between personal achievement and well-being (*r* = 0.203, *P* = 0.026). Other variables, such as self-control, sociability and emotionality, did not correlate significantly with personal achievement. We found no significant correlation between emotional intelligence factors of well-being, self-control, emotionality and sociability, and other burnout variables.

**Table 5:** Relationship between the subscale of trait emotional intelligence and burnout derived from the abbreviated Maslach Burnout Inventory

Global score of TEI	Emotional exhaustion		Depersonalisation		Personal achievement	
	Correlation	*P*-value	Correlation	*P-*value	Correlation	*P-*value
Well-being	0.074	0.419	−0.150	0.103	0.203	0.026
Self-control	−0.012	0.900	0.067	0.466	0.022	0.808
Emotionality	0.086	0.350	0.051	0.585	−0.016	0.865
Sociability	0.121	0.187	0.022	0.814	0.156	0.089

TEI, trait emotional intelligence.

## Discussion

Occupational burnout has been explored in the Arabian Gulf countries, but limited studies have simultaneously examined whether there is a link to temperament. To fill the literature gap, this study aimed to quantify how first-year residents self-reported various indicators of occupational burnout and emotional intelligence, how specific dimensions of emotional intelligence are related to burnout and whether specific sociodemographic characteristics are associated with burnout.

Among the 120 first-year residents who consented to participate, the majority were female (76.7%). The recent modernisation in Oman increased women's empowerment and participation in the labour force. As a result, the healthcare infrastructure in Oman is predominantly female. The gender distribution in this study echoes this. Studies have indicated that occupational burnout takes a toll on all levels of the medical profession, and those who experience burnout are likely to continue to do so after qualifying as a physician.^4^ This study found that 25.8% of the participants had a high burnout status. The present rate appears to be in the lower range compared with the global prevalence of burnout, ranging from 7.0 to 75.2%.^24^

It may be relevant here to speculate on a relatively lower rate of burnout prevalence found in this study compared with the global range. There may be specific cultural factors for Oman and the structure of the Omani residency programme that may have influenced the way burnout is reported among resident physicians. In traditional Omani society, psychological distress is often less openly acknowledged or supported, as revealing such feelings can be viewed as something that violates social modesty or constitutes culturally devalued conduct. Oman is a collective society where individuals may prioritise group harmony and adherence to social norms over personal emotional expression. This cultural tendency could contribute to the underreporting of burnout. Additionally, the OMSB programme, which fosters a structured and close-knit learning environment, can provide additional support systems to mitigate burnout, although the reluctance to disclose psychological distress remains a potential factor influencing the reported prevalence. Other factors that could contribute to the relaxed attitude are that medical education is universally free for Omani nationals, and jobs for graduating physicians in healthcare service literacy are guaranteed.

The present study showed that the mean emotional intelligence trait of all residents was 4.77 (±0.64), which is less than the average score reported by Al Huseini et al^18^ and other studies.^19,25^ There were no significant differences in the emotional intelligence of the trait and its subscales among residents who differed in specialties, age and socioeconomic status, similar to the findings of many international studies.^25–28^ Analysing the various correlates of occupational burnout could shed light on which factors could prevent high burnout among medical professionals. Data from the current study indicated a significant positive correlation between personal achievement and well-being: participants who reported a higher sense of personal achievement also reported increased well-being. However, there were no significant correlations between other indices of emotional intelligence and other subscales of occupational burnout. This partially supports the hypothesis of the study, as there is a significant correlation between occupational burnout and emotional intelligence. This confirms the impact of well-being on burnout, which was previously demonstrated in a study by Nastasa and Farcas.^29^ Several factors may explain the absence of a significant link between emotional exhaustion, depersonalisation and emotional intelligence in our study. One possible explanation is that our study was conducted among first-year medical residents at the beginning of their training, which may have influenced the outcome. Previous research suggests that residents in the early stages of their training experience less pressure compared with those who are further along in their residency training.^30^

Furthermore, the higher proportion of women in our sample could explain the lack of connection between emotional exhaustion and depersonalisation with emotional intelligence, in terms of burnout experience and manifestation. Other unmeasured factors, such as resilience, perceived job control and social support, may act as mediators in the relationship between emotional exhaustion and burnout. Future research could explore these factors as potential moderators in the relationship between emotional intelligence and burnout dimensions, such as emotional exhaustion and depersonalisation.

There are likely to be some challenges in emotional intelligence as it pertains to burnout. Emotional intelligence refers to the ability to perceive, understand, manage and utilise emotions effectively in oneself and others.^31^ It encompasses skills such as emotional awareness, empathy, self-regulation and social skills. The construct of emotional intelligence has gained widespread acceptance since Daniel Goleman's influential book *Emotional Intelligence*, published in the mid-1990s. Research supports its validity, showing that emotional intelligence can influence various aspects of personal and professional life, including job performance, leadership and interpersonal relationships.^32^ Several assessments have been developed to measure emotional intelligence, including the TEIQue-SF, which is currently used. However, there are ongoing debates and critiques regarding the definitions and measurement of emotional intelligence. Some argue that it overlaps with other psychological constructs or that current emotional intelligence measures may lack precision.^33^

Some covariates of burnout, including age, experience, specialisation, gender and marital status, have been suggested to play a role in the expression of occupational burnout.^7,8^ The findings from this study demonstrate a significant correlation between age and the depersonalisation and personal achievement subscales. The older participants reported higher rates of depersonalisation and lower personal achievement, often leading to burnout. Previous research demonstrated similar findings, suggesting that older professionals with more work experience have higher chances of experiencing occupational burnout.^34^ However, other studies have found a bimodal relationship between age and burnout. A study by Marchand et al^35^ found that age was positively correlated with burnout until 30 years of age, negatively correlated up to 55 years of age and then again positively correlated above 55 years of age. This highlights a separate assumption and suggests that burnout syndrome is influenced by different stages of life rather than age alone. Previous studies have suggested that occupational stress tends to have a commutative pattern. Thus, there is an incremental increase in stress as one progresses in one's medical career.^36^ This would imply that burnout is not a pre-existing disposition before entering medical school; instead, it is acquired during medical training. Studies in Oman have suggested that, compared with other specialties in art and science subjects, medical students are more prone to experience poor coping.^37^

This study also noted that marital status influences burnout, and married participants reported higher burnout rates, specifically in emotional exhaustion. This could be explained by work–family conflicts, especially since most of the sample was female. In particular, in many countries, including Oman, married women were often deemed responsible for housekeeping and caregiving, which therefore might increase the number of work–family conflicts and, as a result, increased levels of burnout, especially for women with highly demanding jobs. In other studies, marital status is often noted as a significant covariate in the trajectory of burnout^6,7^ and essential for work–life balance.^38^ A study in Turkey suggested that married women working in emergency departments in Turkey appeared to be at high risk of poor coping.^39^ In Oman, tradition dictated gender segregation, with women taking the role of homemakers.^40^ In recent years, female empowerment, mainly by including female students in education, has resulted in women entering the modern economy. However, tradition still means they have expected roles such as homemaking and motherhood. Such conflicting expectations are likely to mean that women in the labour force have to juggle their newly acquired role as a doctor and their traditional role as housewives.^41^ This could explain why marriage may be a larger factor for burnout among Omani women doctors than their male counterparts,^42^ although not all studies share this view.^43^

### Limitations

Psychosocial studies such as this tend to have various limitations because the concepts investigated need central features. Similarly, the validity of those concepts should be clarified when used in different populations and linguistic groups. Second, the generalisation of this study is likely to be hampered by the fact that it used a cross-sectional approach, which can present suboptimal robustness. Third, pre-existing emotional disorders and different personality types tend to shape one's reactivity to burnout and subjectivity when expressing emotional intelligence, which has not been accounted for in this study. Fourth, the present study is likely to be marred with selection bias; that is, participants were recruited during a workshop. This implies that those who did not attend might differ in important ways (e.g. in levels of burnout or emotional intelligence), leading to results that may not be generalisable to all residents in the OMSB programme. Future studies could try to reach residents outside of the workshop scenario. Fifth, although the study explores various factors that could be associated with burnout and emotional intelligence, future studies could also include variables such as working hours, work–life balance and socioeconomic status. In addition, to attempt to circumvent some of the limitations mentioned above, longitudinal components are needed in future research to track burnout and emotional intelligence over time, to better understand their relationship.

In conclusion, among the first-year Omani resident physicians included in this study, the prevalence of burnout was 25.8%, with high numbers experiencing burnout related to emotional exhaustion and depersonalisation. Age and marital status were the sociodemographic factors most significantly associated with reported burnout. The take-home message from this study is that married female residents should be closely monitored for burnout, with provisions made to improve their work–life balance.
